# Management of Glaucoma in Cytomegalovirus Uveitis: A Retrospective Case Series of Surgical and Medical Approaches

**DOI:** 10.7759/cureus.76946

**Published:** 2025-01-05

**Authors:** Low Chun Heng, Nor Idahriani Muhd Nor

**Affiliations:** 1 Ophthalmology, Hospital Kuala Lumpur, Kuala Lumpur, MYS

**Keywords:** cytomegalovirus (cmv), glaucoma drainage implants, glaucoma-filtering surgery, glaucoma treatment, hypertensive anterior uveitis, minimal invasive glaucoma surgery, secondary open angle glaucoma

## Abstract

Cytomegalovirus (CMV) is a double-stranded (dsDNA) virus of the herpesvirus family. Serological tests reveal signs of previous exposure to it in 40% to 100% of the general population. CMV anterior uveitis (AU) is the most common form of ocular manifestation of CMV in immunocompetent individuals. Clinically, it manifests mainly as anterior chamber (AC) inflammation, iris atrophy, and elevated intraocular pressure (IOP). In this study, we presented four cases of CMV AU with high IOP requiring different treatment modalities to control the IOP.

All patients underwent AC paracentesis, and the aqueous sample sent for polymerase chain reaction (PCR) showed CMV DNA. They were treated with ganciclovir ophthalmic gel 0.15% for the infection. For IOP control, patients underwent different surgeries, namely microinvasive glaucoma surgery (MIGS) with XEN implant, augmented trabeculectomy (AT), glaucoma drainage device implantation (GDI), and transscleral cyclophotocoagulation (TSCPC). Patients were then followed up for a period ranging from six months to three years post-intervention to monitor for evidence of recurrence, IOP control, number of topical antiglaucoma medications required, and progression of glaucoma as evidenced by optical coherence tomography (OCT) retina nerve fiber layer (RNFL) and Humphrey visual field (HVF).

## Introduction

Cytomegalovirus (CMV) anterior uveitis (AU) is the most common ocular manifestation of CMV in immunocompetent individuals [1]. Acute CMV AU typically presents as Posner-Schlossman syndrome (PSS) in Asian men aged between 30 and 50 years [2]. The disease is characterized by recurrent acute episodes of mild, unilateral, non-granulomatous AU with severely elevated intraocular pressure (IOP) [3]. Patients usually present with symptoms such as blurred vision, ocular pain, and photophobia, lasting from a few hours to several weeks and resolving spontaneously. Clinical signs include elevated IOP, anterior chamber (AC) inflammation, iris atrophy, endotheliitis, and, importantly, circinate keratic precipitates (KPs) [3]. Although the disease is typically benign, frequent or prolonged acute episodes can lead to glaucomatous optic neuropathy.

Diagnosing CMV AU clinically can be challenging, as it shares overlapping clinical signs with other, more common infectious causes of AU, such as herpes simplex virus (HSV) AU and varicella zoster virus (VZV) AU [4]. Therefore, a high degree of suspicion and an accurate diagnosis are crucial, as CMV AU requires different antiviral therapy. Once suspected, an aqueous sample should be obtained via paracentesis and analyzed to differentiate among CMV, HSV, and VZV. Diagnostic tests for CMV include reverse transcriptase PCR (RT-PCR) and the Goldmann-Witmer coefficient (GWc). The RT-PCR test directly detects the viral genome load, while the GWc test compares the level of CMV antibodies in the aqueous humor and serum [5].

Early diagnosis and prompt treatment with antiviral therapy are essential to prevent complications such as cataracts, glaucoma, endothelial dysfunction, and permanent corneal damage [6]. Human CMV responds well to ganciclovir and valganciclovir. To date, no clear guidelines exist for the administration or duration of antiviral treatment for CMV AU. The treatment can be administered orally, intravenously, or topically.

Reducing IOP during an acute attack is equally important to prevent glaucomatous optic neuropathy. Topical IOP-lowering medications, such as beta-blockers, carbonic anhydrase inhibitors, alpha-agonists, and prostaglandin analogs, can be used as first-line treatments. Systemic therapies, such as oral carbonic anhydrase inhibitors, intravenous mannitol, and oral glycerol, can also serve as temporizing measures. If all of the above fail, glaucoma surgeries, including microinvasive glaucoma surgery (MIGS), augmented trabeculectomy (AT), glaucoma drainage device implantation (GDI), or transscleral cyclophotocoagulation (TSCPC), may be helpful [7].

## Case presentation

Case study 1

**Figure 1:**
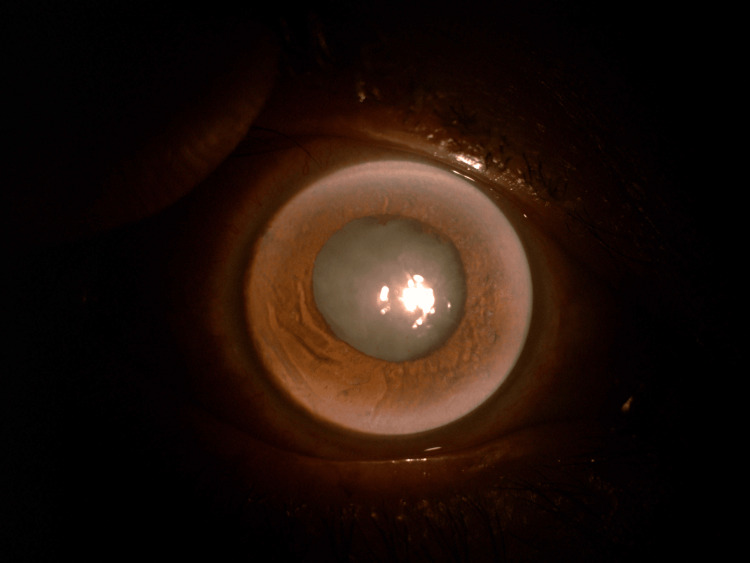
Anterior segment of the RE during initial presentation, showing generalized corneal edema (due to high IOP) with brown pigment on the inferior cornea and anterior lens capsule. RE, right eye; IOP, intraocular pressure

**Figure 2:**
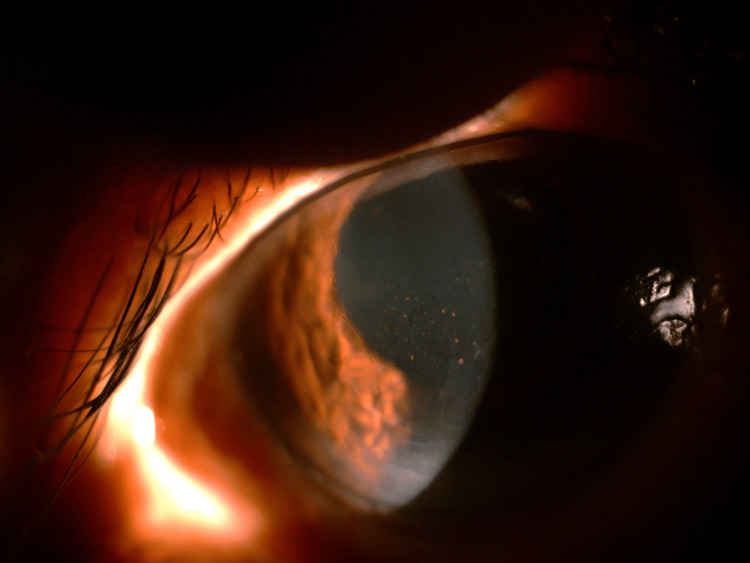
Slit-lamp image showing inferior KPs, indicative of endothelial involvement commonly seen in CMV AU. CMV, cytomegalovirus; AU, anterior uveitis; KPs, keratic precipitates

A 34-year-old male with no medical illness was referred to our clinic with sudden onset of right-sided headache, right eye (RE) redness, and photophobia for the past five days. Upon further questioning, he recalled episodes of similar presentations, each lasting a few days and resolving spontaneously. Visual acuity of the RE was 6/36 at presentation, and IOP was 46mmHg. RE AC (Figure 1 and Figure 2) revealed conjunctival injection, hazy cornea with AC cells of 1+, brown pigment over the inferior cornea, and anterior lens capsule. RE cup-disc ratio (CDR) was 0.3 with no evidence of posterior uveitis.

He was initially treated for pigment dispersion syndrome with a differential diagnosis of uveitic glaucoma and started on G prednisolone acetate every 2 hours, along with topical antiglaucoma medications such as timolol, brimonidine, dorzolamide, and bimatoprost. Uveitic workup for both infectious (tuberculosis and syphilis) and non-infectious causes was negative. Due to the hazy corneal periphery, the patient was counseled for RE AC paracentesis to aid diagnosis and TSCPC to control the IOP.

An aqueous sample was sent for CMV, HSV1/2, and VZV RT-PCR. The results were positive for CMV. Ganciclovir ophthalmic gel 0.15%, five times per day, was started with tapering. Post-TSCPC, his IOP remained around 18-24 mmHg for a period of nine months on all four topical antiglaucoma medications. Visual acuity of RE was 6/24 and ph was 6/12.

After nine months, he presented with another episode of increased IOP despite no reactivation of CMV AU (quiet AC). At this point, he was on ganciclovir ophthalmic gel 0.15% once daily. RE IOP on presentation was 43. He underwent RE uncomplicated phacoemulsification and intraocular lens (IOL) implantation with repeat inferior 270° TSCPC. Post-operation, his IOP ranged from 16 to 22 mmHg on timolol eye drops. Throughout his 18 months of follow-up, there was no evidence of glaucomatous changes.

Case study 2

A 59-year-old male with no medical illness was referred to our center for left eye (LE) AU with high IOP. He initially presented to a prior hospital for recurrent episodes of LE redness and ocular pain. Uveitic workup for infectious (tuberculosis and syphilis) and non-infectious causes was negative. Topical prednisolone acetate, timolol, and latanoprost were started. His IOP remained stable at 17-21 mmHg. During his last visit, he was having reactivation of AU with uncontrolled IOP despite maximal topical and systemic antiglaucoma treatments.

**Figure 3:**
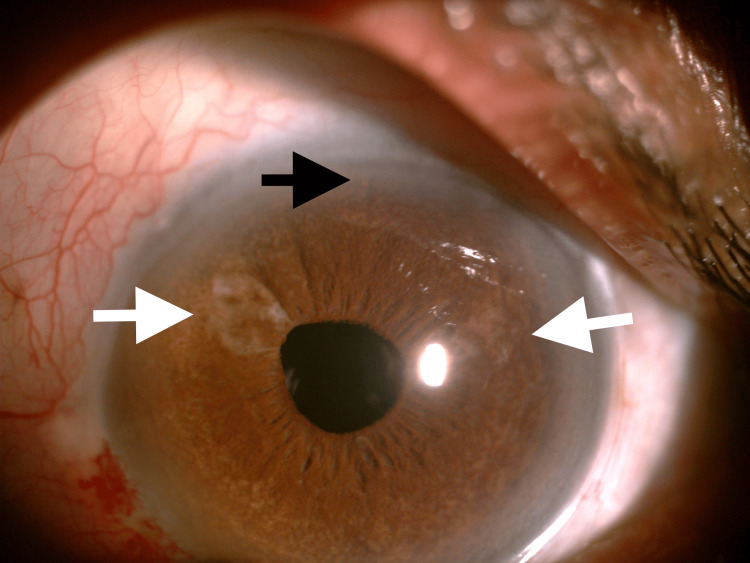
Anterior segment of LE showing sectoral iris atrophy at 2 o'clock and 10 o'clock positions (white arrows), suggestive of viral uveitis. Also, note the presence of the XEN implant at the 12 o'clock position (black arrow). LE, left eye

**Figure 4:**
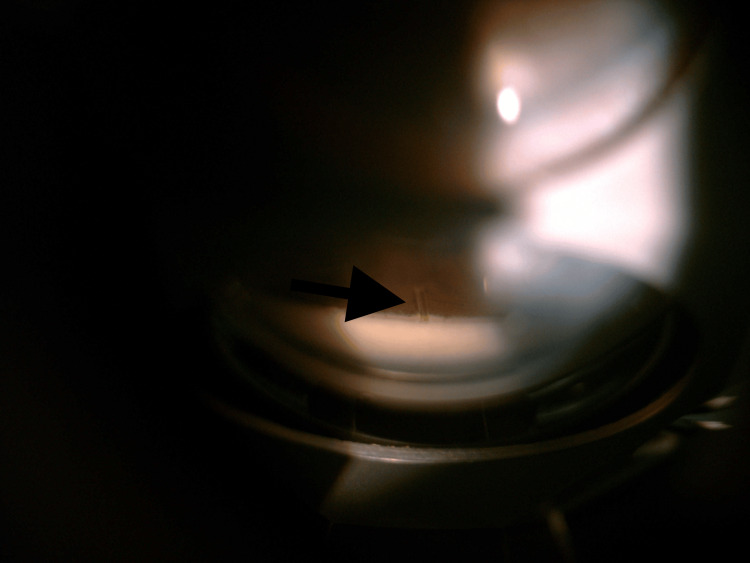
Gonioscopy of the LE post-phacoemulsification, IOL implantation, and XEN implantation showing the XEN implant in situ. The XEN implant creates a new drainage pathway for aqueous humor to flow from the AC to the subconjunctival space, thereby reducing IOP. AC, anterior chamber; LE, left eye; IOP, intraocular pressure; IOL, intraocular lens

**Figure 5:**
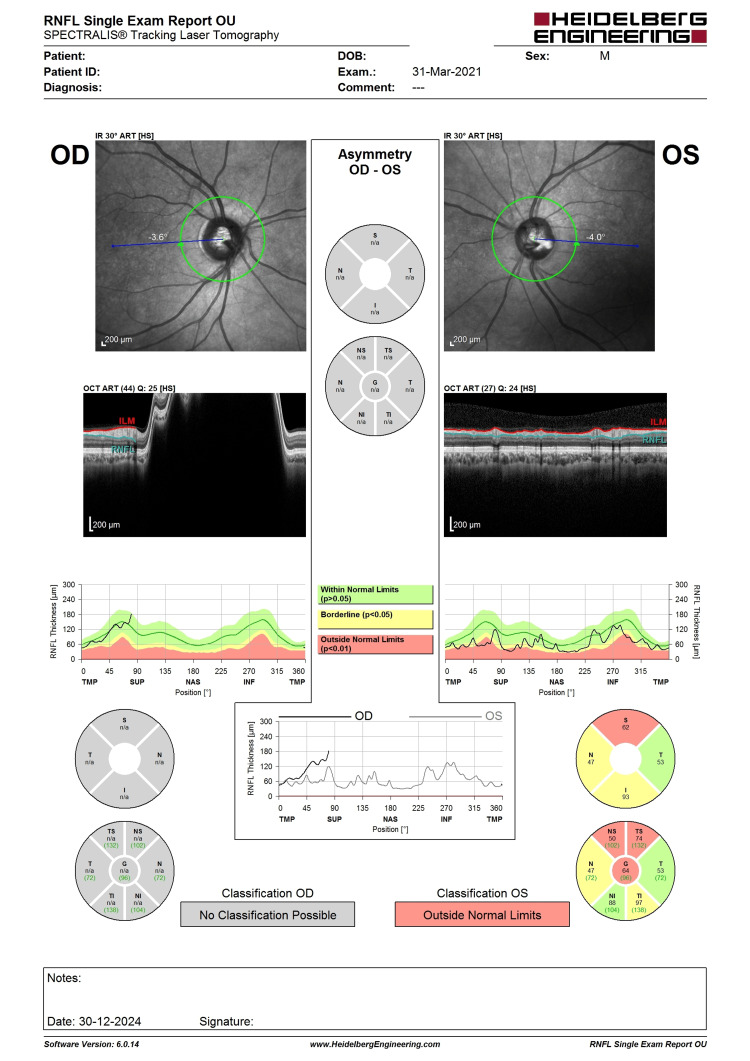
OCT RNFL before LE phacoemulsification, IOL implantation, and XEN implantation. Note the LE central RNFL thickness of 64 µm with evidence of superior RNFL thinning (highlighted in red). LE, left eye; IOL, intraocular lens; OCT, optical coherence tomography; RNFL, retina nerve fiber layer

**Figure 6:**
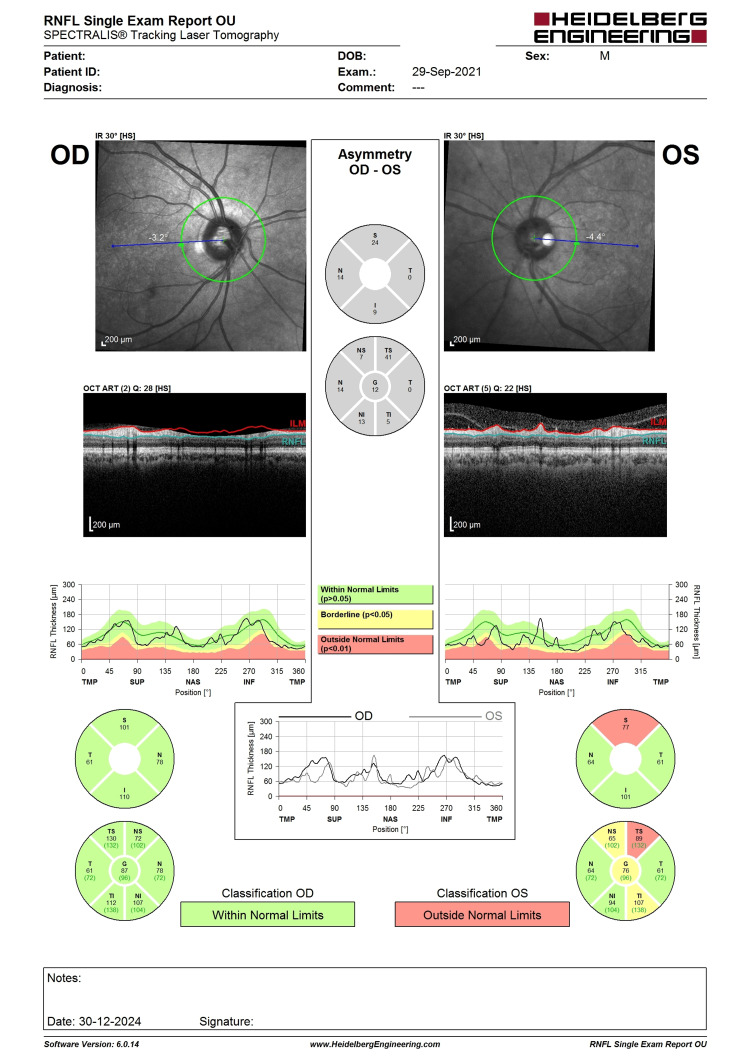
Six-month post-intervention OCT RNFL showing a similar pattern of superior RNFL thinning with central RNFL thickness of 76 (not worsening). The improvement could be the result of cataract extraction improving the signal strength. OCT, optical coherence tomography; RNFL, retina nerve fiber layer

**Figure 7:**
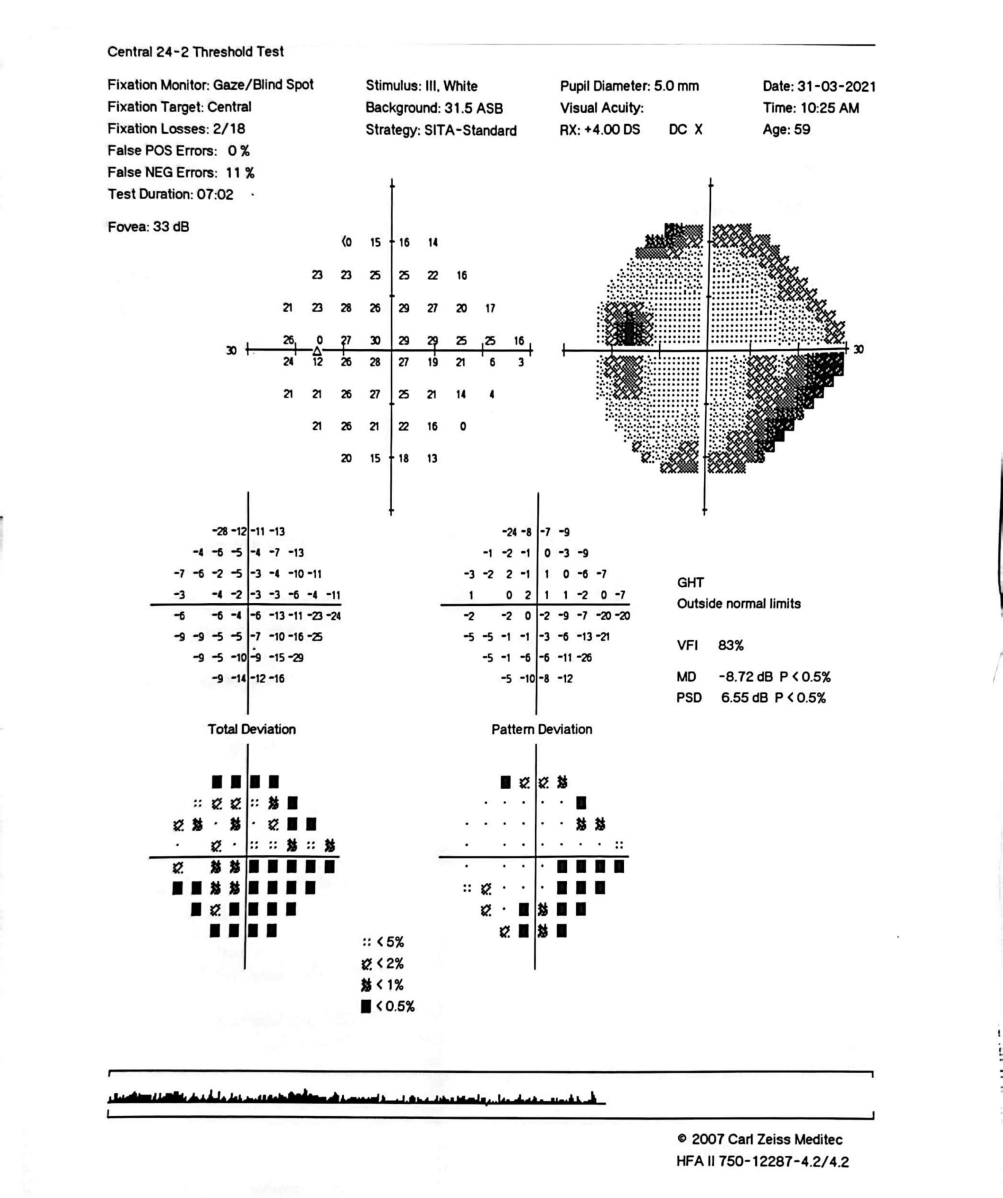
Pre-intervention HVF showing inferior arcuate scotoma (in pattern deviation) correlating well with superior RNFL thinning from OCT (Figure 5). Also, note the VFI of 83%. VFI, visual field index; HVF, Humphrey visual field; OCT, optical coherence tomography

**Figure 8:**
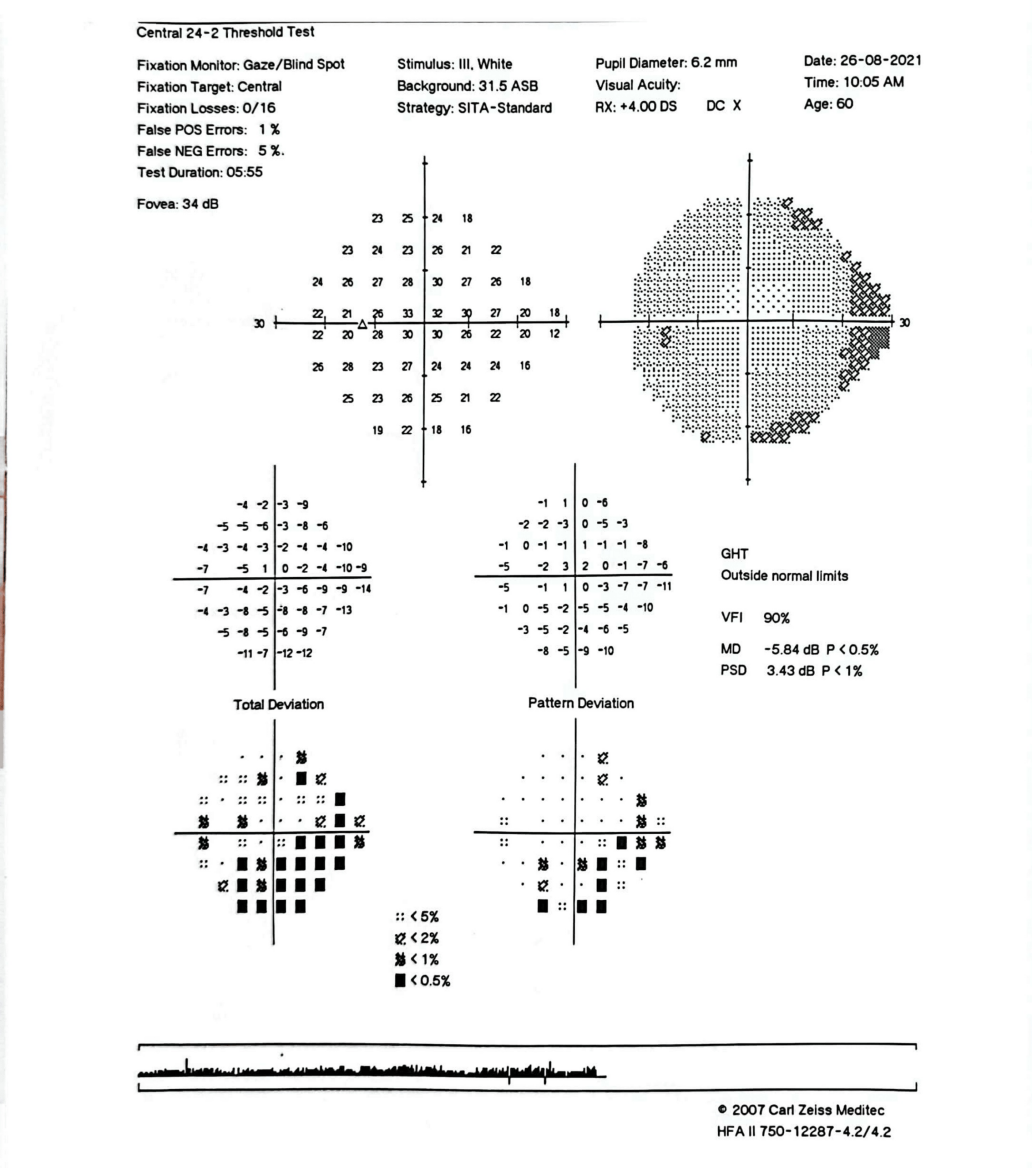
Six-month post-intervention HVF showing inferior arcuate scotoma (similar visual field defect). The slight improvement of VFI to 90% could be due to cataract extraction. VFI, visual field index; HVF, Humphrey visual field

Initial review at our center showed visual acuity of 6/12 in the LE and an IOP of 30 mmHg while on topical prednisolone acetate every 2 hours, timolol, dorzolamide, brimonidine, latanoprost, and oral acetazolamide 250 mg QID. LE AC examination (Figure 3) showed sectoral iris atrophy, AC cells 1+ with the presence of old KPs on the cornea, and lens nucleosclerosis 2+, with a CDR of 0.6. In view of the high suspicion of viral AU, as evidenced by sectoral iris atrophy, recurrent episodes of AU with high IOP, and the presence of KPs, AC paracentesis was performed. An aqueous sample was sent for HSV1/2, CMV, and VZV RT-PCR, and the result was positive for CMV. Ganciclovir ophthalmic gel 0.15%, five times daily, was started with tapering. A joint decision was made for LE phacoemulsification with IOL and XEN implantation (Figure 3 and Figure 4) in view of moderately elevated IOP. Post-operatively, his LE vision was 6/9 and IOP remained at 10-14 mmHg without any topical antiglaucoma medications. He showed no glaucoma progression as evidenced by serial optical coherence tomography (OCT) retina nerve fiber layer (RNFL) and Humphrey visual field (HVF) (Figures 5-8). There was no evidence of reactivation.

Case study 3

A 46-year-old male with underlying hypertension was referred to our center for LE AU with high IOP. At a previous center, he had multiple clinic visits for LE redness, blurred vision, and ocular pain. Each episode required treatment with topical prednisolone acetate, timolol, brimonidine, and latanoprost. Prior to referral to our center, he was admitted for recurrent LE AU with a high IOP of 42 mmHg and was started on topical prednisolone acetate, timolol, brimonidine, latanoprost, dorzolamide, and oral acetazolamide 250 mg QID. Despite treatment, his IOP ranged from 15 to 27 mmHg.

Initial review at our center showed visual acuity of LE as 6/9, with an IOP of 28 mmHg on topical prednisolone acetate, timolol, brimonidine, latanoprost, dorzolamide, and oral acetazolamide 250 mg QID. LE AC examination revealed an AC cell of 2+ and old pigmented KPs at the inferior half of the cornea. The CDR was 0.4. He was treated for LE AU with ocular hypertension and was counseled for staged surgery of LE phacoemulsification/IOL implantation/TSCPC first, followed by glaucoma filtering surgery after inflammation resolved. He agreed.

**Figure 9:**
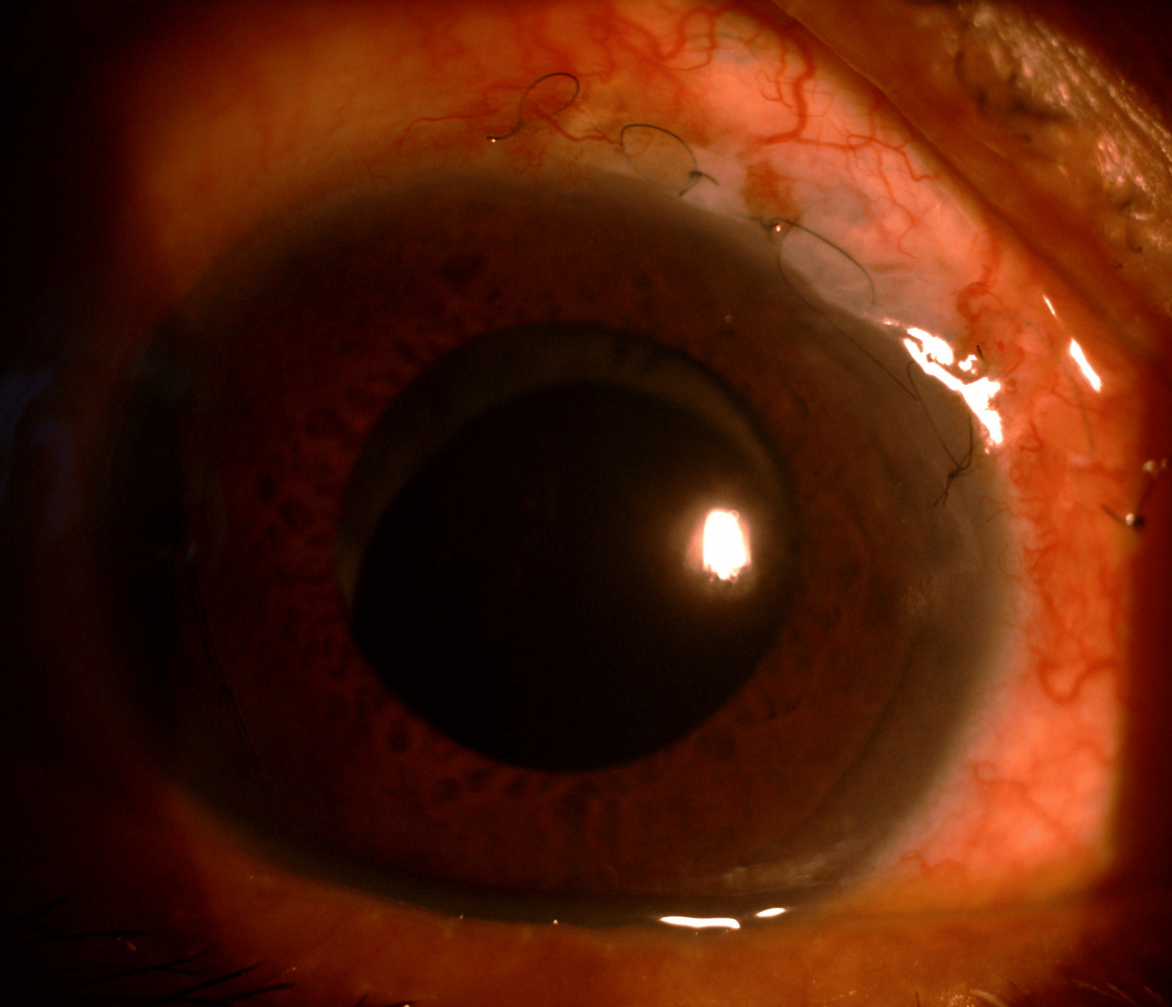
Anterior segment of LE post-trabeculectomy + mitomycin C showing superotemporal bleb. LE, left eye

**Figure 10:**
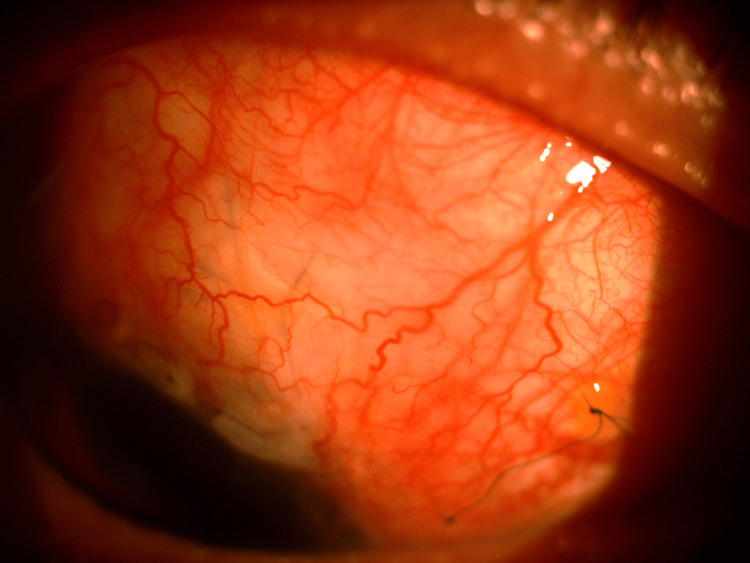
A closer view of the LE superotemporal quadrant showing a functional bleb extending from 11 o'clock to 2 o'clock. LE, left eye

**Figure 11:**
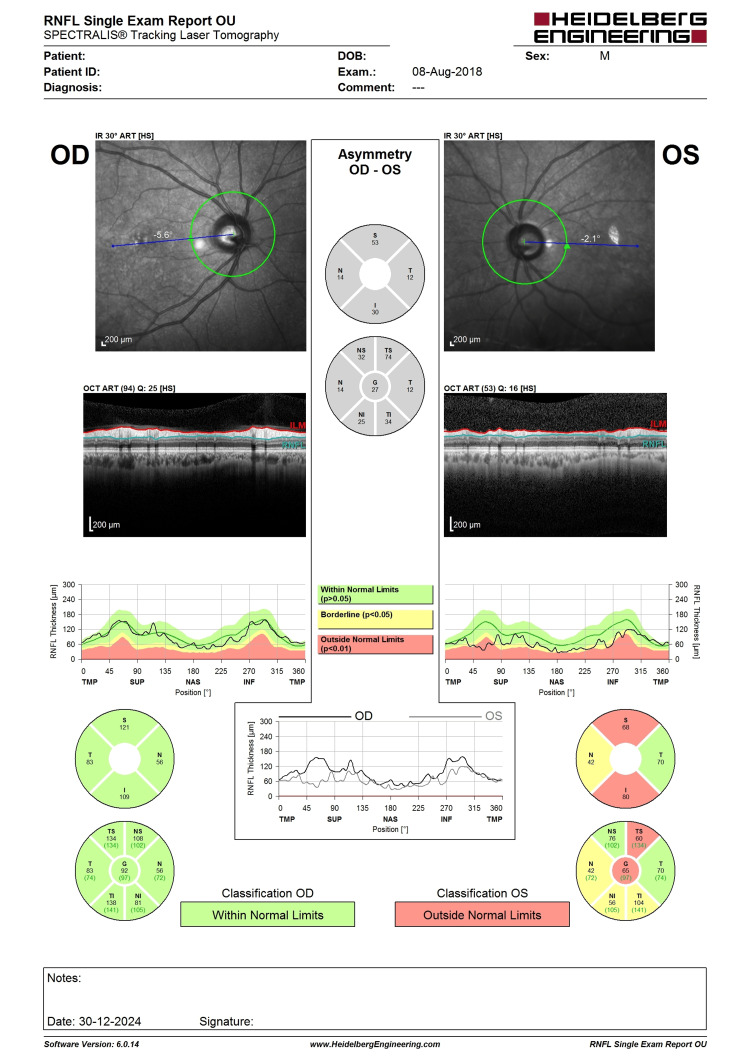
Pre-trabeculectomy OCT RNFL showing LE CDR of 0.7 with central RNFL thickness of 65. There was associated superior and inferior RNFL thinning (highlighted in red). OCT, optical coherence tomography; RNFL, retina nerve fiber layer; LE, left eye; CDR, cup-disc ratio

**Figure 12:**
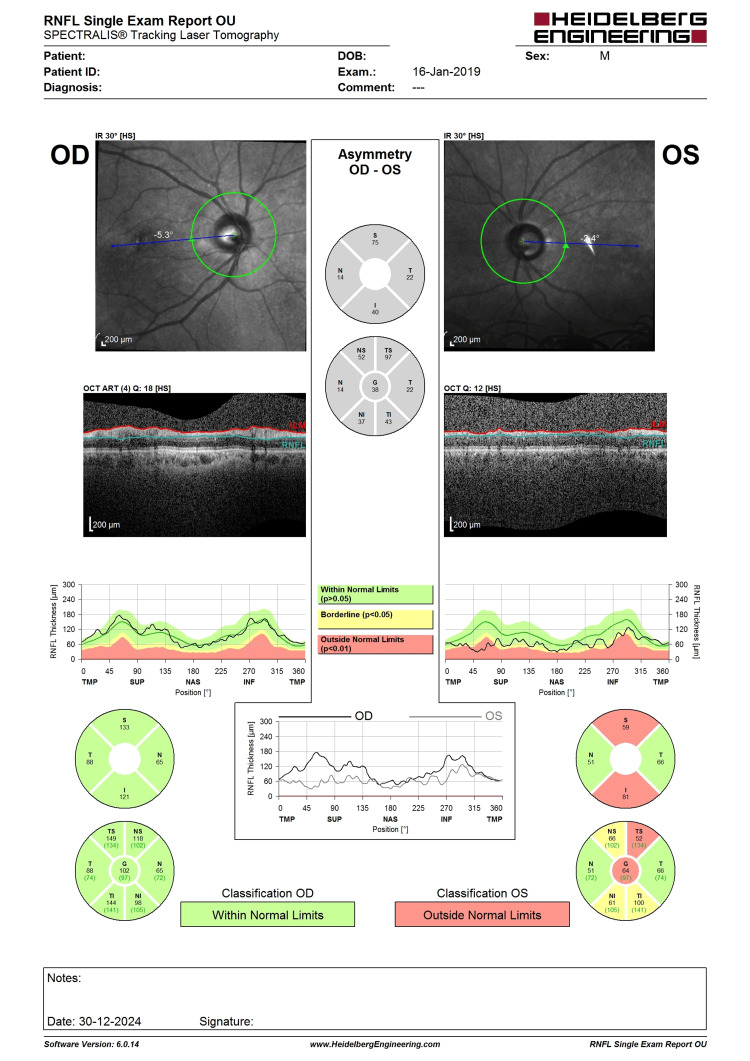
Five-month post-trabeculectomy OCT RNFL showing a similar pattern of superior and inferior RNFL thinning with central RNFL thickness of 64. OCT, optical coherence tomography; RNFL, retina nerve fiber layer

**Figure 13:**
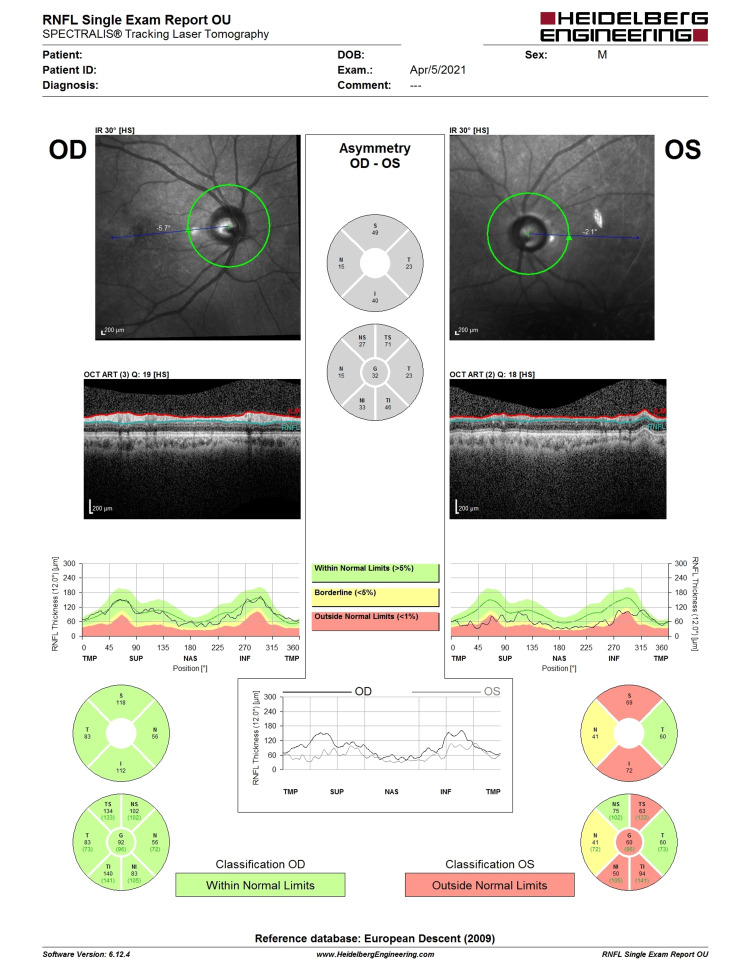
OCT RNFL two years eight months post-trabeculectomy showing lack of glaucoma progression despite bleb failure. OCT shows a central RNFL thickness of 60 with similar superior and inferior thinning. OCT, optical coherence tomography; RNFL, retina nerve fiber layer

He underwent LE uncomplicated phacoemulsification/IOL implantation, aqueous tap, and inferior 180° TSCPC. RT-PCR of the aqueous revealed the presence of CMV DNA, and ganciclovir ophthalmic gel 0.15% was started. Post-intervention, his IOP remained high (27-44 mmHg) on four topical IOP-lowering agents, with progressive enlargement of the CDR from 0.4 to 0.7. LE trabeculectomy with mitomycin C 0.03% was performed (Figure 9 and Figure 10). Post-operatively, the patient remained eyedrop-free with stable IOP (14-22 mmHg) for 15 months until he defaulted. Two years post-trabeculectomy, he presented again with high IOP in the LE and a localized bleb. LE IOP was 27-38 mmHg during multiple clinic visits. The patient refused bleb needling and was started on topical timolol, brimonidine, and dorzolamide. He defaulted again after a year of follow-up, with the last documented LE IOP of 17. There was no evidence of progression of glaucoma after trabeculectomy (Figures 11-13).

Case study 4

**Figure 14:**
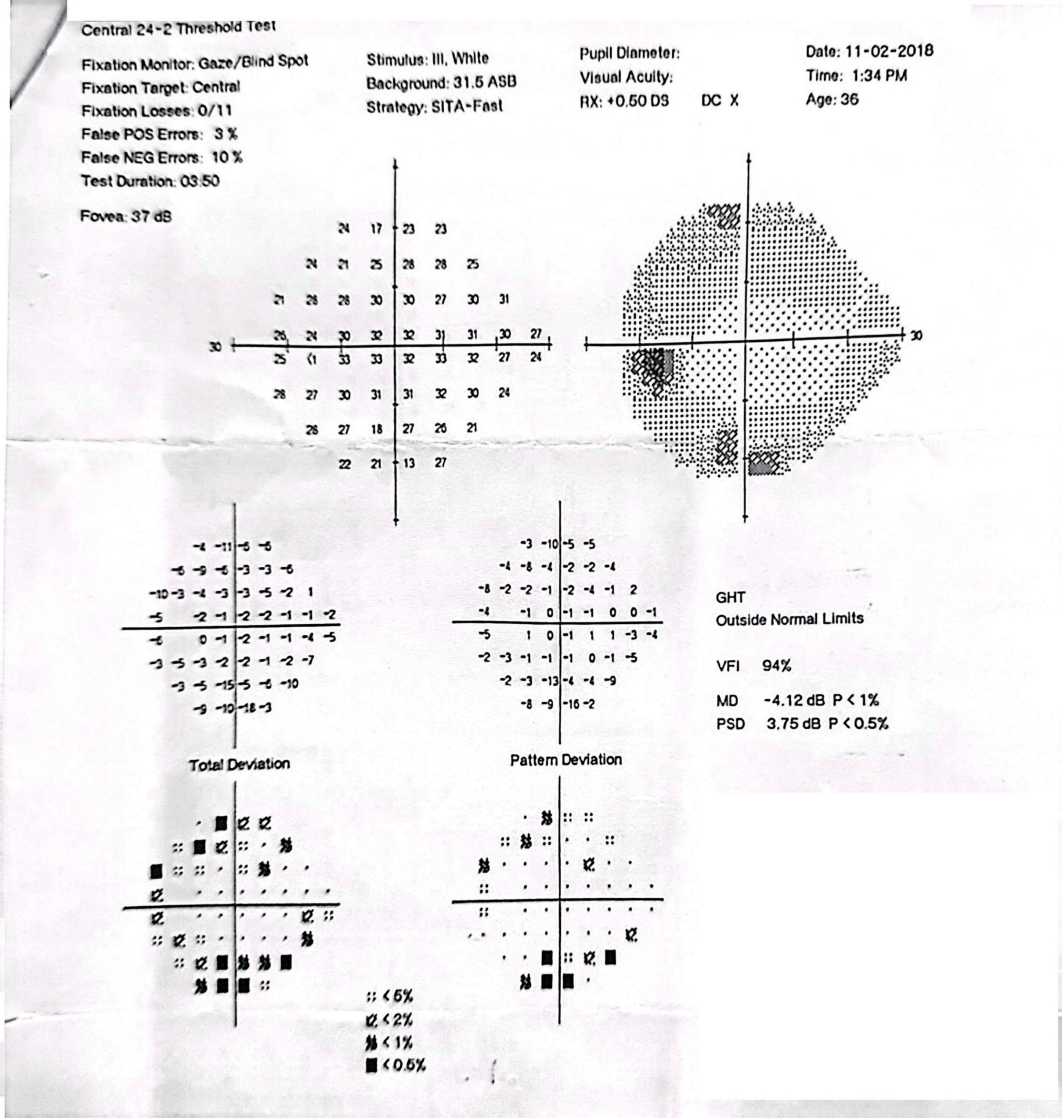
LE HVF dated 2018 from the primary treating hospital showing moderate glaucomatous changes of inferior arcuate scotoma with VFI of 94%. Despite this, the patient refused to be referred for surgical intervention. LE, left eye; HVF, Humphrey visual field; VFI, visual field index

A 41-year-old male with underlying hypertension was referred to our center for LE AU with persistent high IOP. He is a frequent relapser, experiencing about one episode per year for a total of seven years. Each episode was accompanied by LE redness, blurred vision, and ocular pain, with IOP rising to 30-60 mmHg, requiring maximum topical and systemic antiglaucoma medications. Prior to this, he had been repeatedly counseled to be referred for surgical intervention due to glaucomatous changes in his HVF (Figure 14). However, he refused.

**Figure 15:**
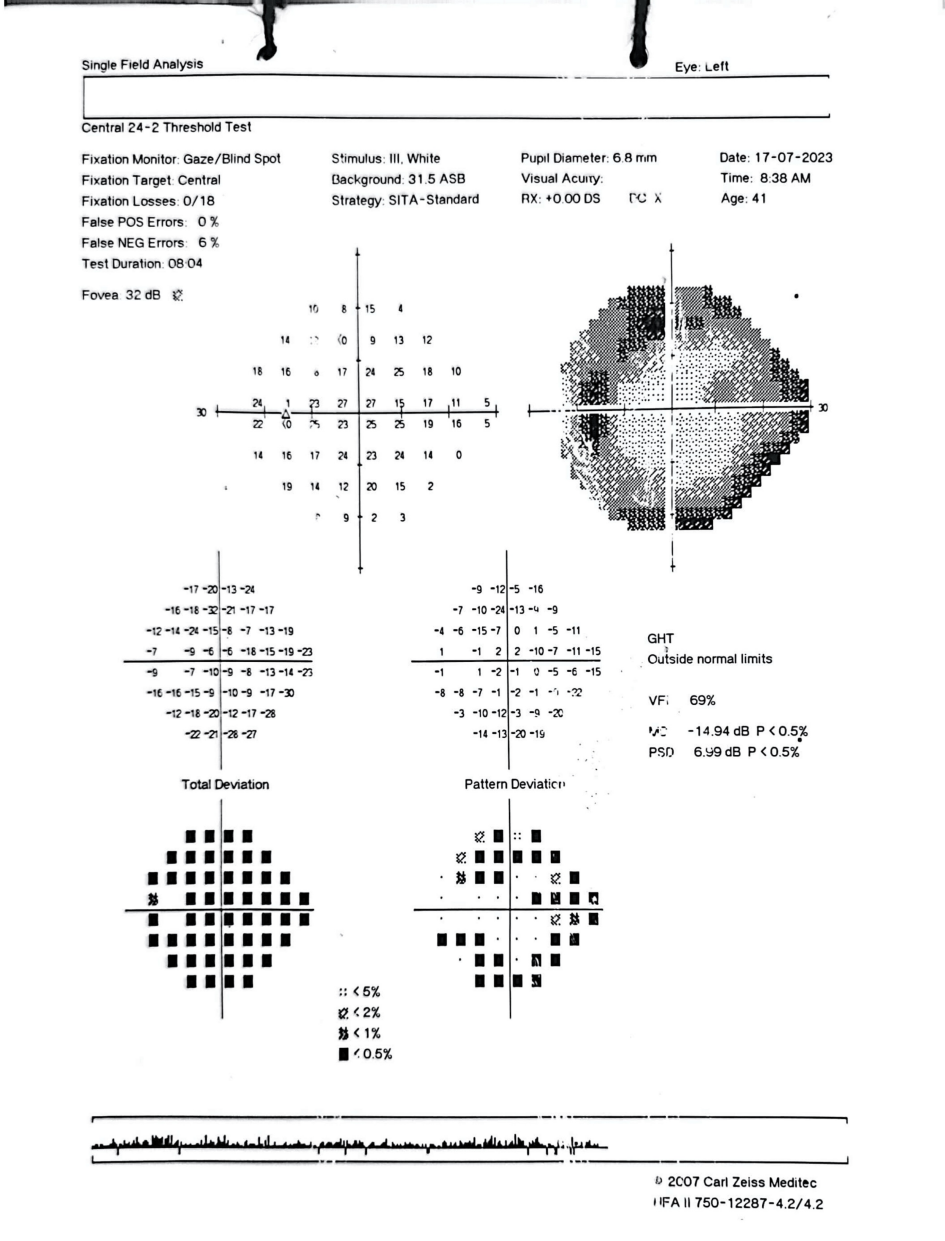
LE HVF five years later after recurrent episodes of AU with high IOP. Note the worsening of VFI to 60% with double arcuate scotoma in pattern deviation. LE, left eye; HVF, Humphrey visual field; VFI, visual field index; IOP, intraocular pressure; AU, anterior uveitis

**Figure 16:**
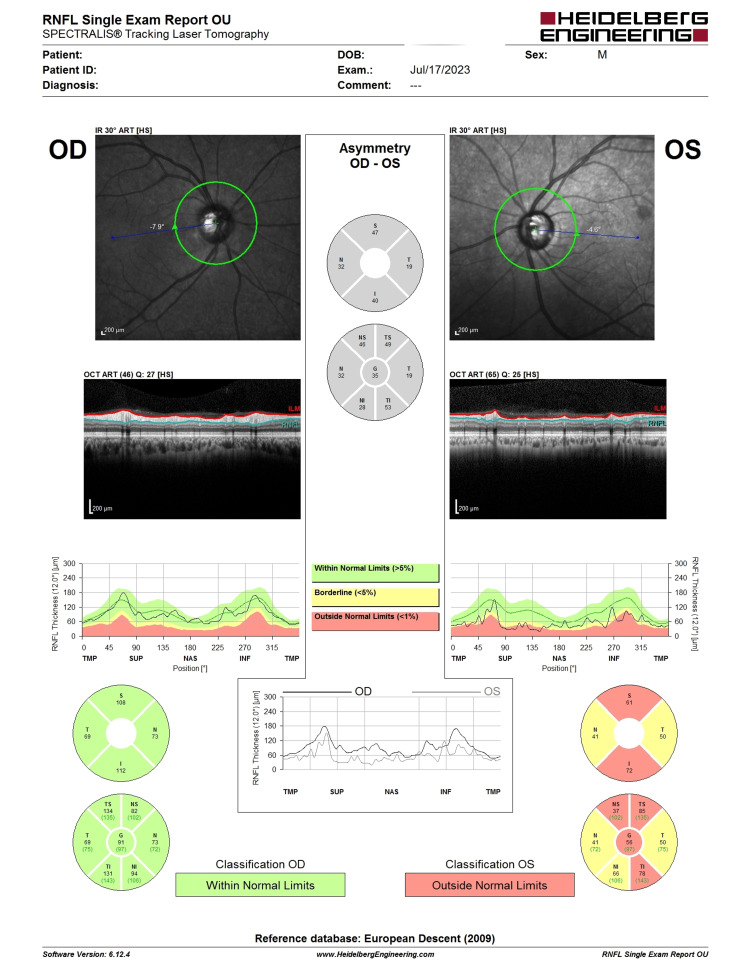
OCT RNFL of the patient showing advanced glaucomatous changes. LE CDR of 0.9 and RNFL thinning 360° (worse in superior and inferior) with a central thickness of 56. OCT, optical coherence tomography; RNFL, retina nerve fiber layer; CDR, cup-disc ratio; LE, left eye

Initial review at our center showed LE visual acuity of 6/18, RAPD negative, with an IOP of 56 mmHg on topical dexamethasone, timolol, brimonidine, dorzolamide, latanoprost, oral acetazolamide 250 mg QID, and syrup glycerol 30 mL TDS. LE AC showed AC cells of 2+, fine KPs centrally, with a white conjunctiva and clear cornea. Fundus examination revealed an LE CDR of 0.9. HVF and OCT RNFL of the LE showed advanced glaucomatous changes (Figure 15 and Figure 16).

**Figure 17:**
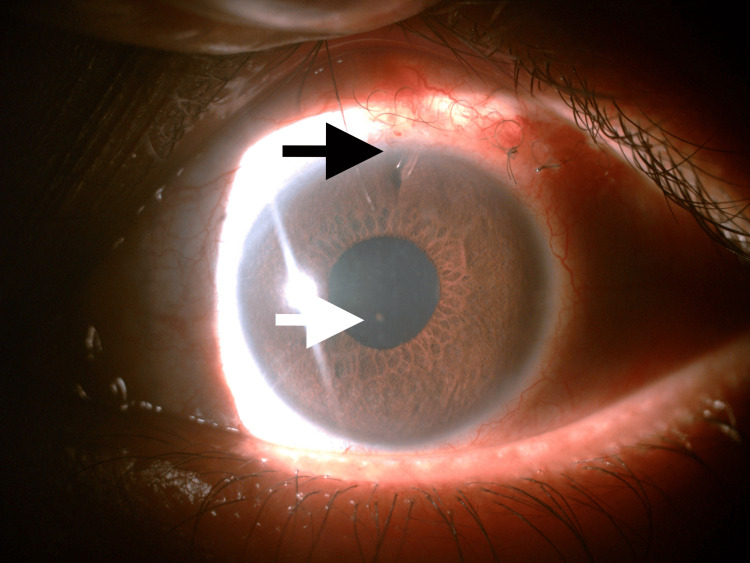
Anterior segment of LE showing few pigmented KPs (white arrow) with Paul tube implant at 12 o'clock position (black arrow). LE, left eye; KPs, keratic precipitates

AC paracentesis was performed to reduce the IOP, and the aqueous sample was sent for RT-PCR, which later revealed the presence of CMV DNA. Ganciclovir ophthalmic gel five times per day was started without delay. In view of advanced glaucoma (CDR: 0.9) with a high risk of failure for filtering surgery (due to frequent relapses), the patient underwent GDI with a Paul tube implant (Figure 17). Six months post-operation, the Prolene stent was removed as his IOP had risen to 30 mmHg. Since then, his IOP has remained stable (7-14 mmHg) without antiglaucoma agents. There was no sign of reactivation of CMV AU, and no progression of glaucoma.

## Discussion

In this case series, patients presented with recurrent episodes of eye redness associated with ocular pain, which resolved either spontaneously or with treatment (Table 1). Ocular examinations revealed high IOP along with evidence of AC inflammation (cells or KPs) and iris atrophy. The primary uveitis workup to investigate non-infectious causes and common infectious causes (syphilis and tuberculosis) was negative in all cases. All patients were treated empirically with topical steroids and IOP-lowering medications. The decision to obtain an aqueous sample for RT-PCR was based on the history of recurrence and clinical signs, including AC cells, iris atrophy, KPs, and high IOP. RT-PCR was chosen due to the availability of the test at our center and the small amount of aqueous sample obtained in all cases.

**Table 1:** Table summarizing patient demographics, presenting symptoms, clinical findings, treatment modalities, and post-operative outcomes. IOL, intraocular lens; CMV, cytomegalovirus; AU, anterior uveitis; KPs, keratic precipitates; LE, left eye; OCT, optical coherence tomography; RNFL, retina nerve fiber layer; CDR, cup-disc ratio; GDI, glaucoma drainage device implantation; TSCPC, transscleral cyclophotocoagulation; IOP, intraocular pressure; RE, right eye

Case	Patient Demographics	Presenting Symptoms	Clinical Findings	Treatment Modalities	Post-operative Outcomes
1	34, male, no medical illness	Recurrent right headache, RE redness, and photophobia	Visual acuity RE: 6/36. RE IOP 46 mmHg anterior segment: conjunctival injection, hazy cornea, AC cell 1+, brown pigment over the inferior cornea, and lens capsule. Posterior segment: OD pink, CDR 0.3.	Medical: Ganciclovir ophthalmic gel five times per day, topical prednisolone acetate, bimatoprost, timolol, brimonidine, and dorzolamide. Laser: TSCPC x 2	RE IOP 16-22 mmHg, on topical timolol OD CDR 0.3, no glaucoma. No reactivation of CMV AU
2	59, male, no medical illness	Recurrent LE redness, ocular pain	Visual acuity LE: 6/12. IOP LE: 30 mmHg anterior segment: conjunctiva white, cornea clear, sectoral iris atrophy at 10 o'clock and 2 o'clock, AC cells 1+, old KPs, lens nucleosclerosis 2+. Posterior segment: OD pink, CDR 0.6	Medical: Ganciclovir ophthalmic gel five times per day, topical prednisolone acetate, bimatoprost, timolol, brimonidine, dorzolamide, oral acetazolamide. Surgical: LE phacoemulsification/IOL + XEN implant	LE IOP 10-14 mmHg, not on any topical antiglaucoma OD CDR 0.6, no glaucoma progression No reactivation of CMV AU
3	46, male underlying hypertension	Recurrent LE redness, blurred vision, ocular pain	Visual acuity 6/9. IOP LE: 28 mmHg anterior segment: conjunctiva white, cornea clear, AC cell 2+, pigmented KPs over endothelium. Posterior segment: OD pink, CDR 0.4 (on presentation), CDR 0.8 before trabeculectomy	Medical: Ganciclovir ophthalmic gel five times per day, topical prednisolone acetate, bimatoprost, timolol, brimonidine, dorzolamide, oral acetazolamide. Surgical: LE phacoemulsification/IOL + TSCPC, LE AT	LE IOP 14-22 mmHg with no topical antiglaucoma for 15 months IOP 27-38 (bleb failure), started on topical timolol, brimonidine, dorzolamide -->IOP 17 OD pink, CDR 0.8, no progression of glaucoma No reactivation of CMV AU
4	41, male underlying hypertension	Recurrent LE redness, blurred vision, ocular pain	Visual acuity LE: 6/18, IOP 56 mmHg anterior segment: conjunctiva white, cornea clear, AC cells 2+, KPs over endothelium. Posterior segment: OD pink, CDR 0.9	Medical: Ganciclovir ophthalmic gel five times per day, topical dexamethasone, bimatoprost, timolol, brimonidine, dorzolamide, oral acetazolamide, oral glycerol. Surgical: LE GDI	LE IOP 7-14 mmHg OD pink, CDR 0.9, no progression of glaucoma No reactivation of CMV AU

All four aqueous samples were positive for CMV DNA PCR, confirming the diagnosis of CMV AU. Treatment was initiated immediately. To date, no clear guidelines are available for the administration of antivirals or the duration of treatment for CMV AU. We prescribed 0.15% ganciclovir ointment five times per day for induction, with a tapering dose for maintenance, considering the availability of the medication at our center and the absence of posterior segment involvement in all four patients. The patients were treated with topical ganciclovir for a duration of one year after active inflammation resolved (no AC cell). Treatment outcomes were favorable, as none of the patients experienced any adverse reactions or reactivation of AU during the follow-up period.

During the course of uveitic glaucoma, medical treatment does not control IOP in a high proportion of cases. This is shown in our study, as all patients did not achieve adequate IOP control with medical therapy alone, necessitating surgical/laser interventions. The choice of initial intervention relies on the degree of inflammation, visual prognosis, and the surgeon's preference.

In this study, we demonstrated that GDI, MIGS, AT, and CPC were effective in managing CMV AU. All forms of intervention showed at least a 50% reduction in IOP, with GDI showing the highest reduction compared to MIGS and AT (Table 2). This is in line with other studies. Ventura-Abreu et al. [8] found a mean reduction in IOP of 30% for GDI compared to 7.5% for AT. In another study, the use of XEN implants had a 72% success rate in uveitic glaucoma, with a mean IOP reduction of 62.5% [9]. CPC, albeit less invasive, is not considered first-line due to its potentially devastating consequences. However, there is increasing interest in using it as a first procedure. In our study, we reported a 59% reduction in IOP with CPC, which is comparable to the 51% reduction found by Ventura-Abreu et al [8].

**Table 2:** Percentage of reduction in IOP for different interventions. Note: In case study 3, post-intervention IOP taken as mean IOP before bleb failure. IOP, intraocular pressure; IOL, intraocular lens; GDI, glaucoma drainage device implantation; TSCPC, transscleral cyclophotocoagulation; MIGS, microinvasive glaucoma surgery; AT, augmented trabeculectomy

Case Study	Intervention	Mean IOP Pre-intervention	Mean IOP Post-intervention	Percentage reduction in IOP
1	TSCPC, repeat TSCPC	46 (4 topical antiglaucoma)	19 (1 antiglaucoma)	59%
2	Phaco/IOL + MIGS (XEN implant)	28 (4 topical antiglaucoma + oral acetazolamide)	12 (no antiglaucoma)	57%
3	AT	36 (4 topical antiglaucoma)	18 (no antiglaucoma)	50%
4	GDI	56 (4 topical antiglaucoma + oral acetazolamide + glycerol)	11 (no antiglaucoma)	80%

The study has some limitations. First, the number of cases included in this series is limited, which may affect the generalizability of the results to a larger population. Second, this research also lacks long-term data. Extended follow-up is necessary to evaluate the durability of IOP control and visual outcomes, especially since some patients may experience recurring or reactivating infections over time.

## Conclusions

The case series underscores the importance of considering CMV as a potential cause of recurrent AU, particularly in patients with high IOP, iris atrophy, and a negative primary uveitis workup. RT-PCR testing of aqueous humor provides a reliable method for diagnosing CMV uveitis, and antiviral treatment, combined with appropriate glaucoma management, can yield positive outcomes. Early detection and intervention are crucial to prevent complications such as glaucoma progression and vision loss.

In our studies, ganciclovir ophthalmic gel 0.15% shows promising outcomes for the treatment of CMV AU. None of the patients experienced side effects or reactivation during the course of treatment. For managing high IOP, GDI shows the highest percentage of reduction in IOP compared to other treatment modalities such as MIGS, AT, and TSCPC. This is in line with existing literature. However, this study has limitations, such as a lack of long-term follow-up and a small number of cases. Future studies are needed to further refine treatment strategies and establish standardized protocols for managing CMV AU in clinical practice.
